# Not all waits are equal: an exploratory investigation of emergency care patient pathways

**DOI:** 10.1186/s12913-017-2349-2

**Published:** 2017-06-24

**Authors:** Dawn Swancutt, Sian Joel-Edgar, Michael Allen, Daniel Thomas, Heather Brant, Jonathan Benger, Richard Byng, Jonathan Pinkney

**Affiliations:** 1 Peninsula Schools of Medicine and Dentistry, University of Plymouth, ITTC Building, Plymouth Science Park, Plymouth, PL6 8BX UK; 2 Department of Computer Science, University of Bath, Bath, UK; 3 Medical School, University of Exeter, Exeter, UK; 4 School of Social and Community Medicine, University of Bristol, Bristol, UK; 5 Faculty of Health and Applied Sciences, University of the West of England, Bristol, UK

**Keywords:** Health service research, Acute care, Emergency admissions, Patient care, Value stream mapping, Emergency department, Patient public involvement

## Abstract

**Background:**

Increasing pressure in the United Kingdom (UK) urgent care system has led to Emergency Departments (EDs) failing to meet the national requirement that 95% of patients are admitted, discharged or transferred within 4-h of arrival. Despite the target being the same for all acute hospitals, individual Trusts organise their services in different ways. The impact of this variation on patient journey time and waiting is unknown. Our study aimed to apply the Lean technique of Value Stream Mapping (VSM) to investigate care processes and delays in patient journeys at four contrasting hospitals.

**Methods:**

VSM timing data were collected for patients accessing acute care at four hospitals in South West England. Data were categorised according to waits and activities, which were compared across sites to identify variations in practice from the patient viewpoint. We included Public and Patient Involvement (PPI) to fully interpret our findings; observations and initial findings were considered in a PPI workshop.

**Results:**

One hundred eight patients were recruited, comprising 25,432 min of patient time containing 4098 episodes of care or waiting. The median patient journey was 223 min (3 h, 43 min); just within the 4-h target. Although total patient journey times were similar between sites, the stage where the greatest proportion of waiting occurred varied. Reasons for waiting were dominated by waits for beds, investigations or results to be available. From our sample we observed that EDs without a discharge/clinical decision area exhibited a greater proportion of waiting time following an admission or discharge decision. PPI interpretation indicated that patients who experience waits at the beginning of their journey feel more anxious because they are ‘not in the system yet’.

**Conclusions:**

The novel application of VSM analysis across different hospitals, coupled with PPI interpretation, provides important insight into the impact of care provision on patient experience. Measures that could reduce patient waiting include automatic notification of test results, and the option of discharge/clinical decision areas for patients awaiting results or departure. To enhance patient experience, good communication with patients and relatives about reasons for waits is essential.

## Background

During recent years hospital emergency departments (EDs), in keeping with all components of the United Kingdom (UK) urgent care system, have been under increasing pressure [1]. This pressure has impacted on the ability of EDs to continue meeting the national standard requiring 95% of patients to be admitted, discharged or transferred within four hours [2]. Whilst all EDs are subject to the same standard, local models of patient care vary substantially. In some hospitals, unscheduled acute care is accessed solely through the ED, and ED staff assess all acute, unplanned arrivals, in a “take-all” model of care. In contrast, other hospitals pre-screen, or filter, patients with less urgent needs through alternative pathways, often not involving the ED [2].

A particular challenge for acute care is patient ‘flow’. The open door policy, coupled with a system that is free at the point of delivery, creates an enticing combination to those who might otherwise have chosen alternative care routes or applied watchful waiting to their health condition. When hospitals are working near to full capacity, high attendance rates reduce patient flow and lead to departmental crowding, as transfer or discharge cannot keep pace with new arrivals. The resulting queuing and waiting puts pressure on staff and resources. This also impacts negatively on the patient experience, because waiting is one of the most important factors determining patient satisfaction [3, 4].

Higginson highlighted the impact of slow patient flow and consequent departmental crowding in UK EDs [5]. He observed ED crowding as a significant international concern, although the extent and solutions were uncertain. One approach, from Lean thinking, has been used to address and improve patient flows in the ED, with positive results [6, 7]. Service improvements in satisfaction and waiting time were achieved by using Value Stream Mapping (VSM) to give an in-depth description of departmental flow, and to address the problem points.

VSM comes from Lean thinking, an approach which is useful in improving departmental and cross-departmental effectiveness because it aims to eliminate or minimise waste in organisation of systems [8]. It has been applied in many healthcare patient settings [9], though its application in the ED, which works under a 4-h target in the UK, is of particular interest and relevance. The fundamental concept is to focus on efficiency of the process in delivering what the consumer requires, not the individual resources, meaning that it is a patient-centred rather than physician-centred approach. VSM maps the patient journey in a times-series, providing detailed observational data of the stages within that journey, with a particular focus on waste and variation in care. The goal is on service improvement. Although VSM has been used successfully in individual EDs to affect improvement [6, 7, 10, 11], this approach has not been fully exploited to compare and contrast variation in patient journey times between different EDs systematically.

This study builds upon previous research that examined wait management and patient flow in EDs and aimed to shorten patient waiting times and length of total visit time [12–14]. We planned to examine how organisational differences in care affect patient waits; an issue that is central to the on-going debate around standardisation of hospital care to improve patient flow [15]. The importance from the patient perspective is how their journey time through their hospital visit is managed. We undertook an exploratory investigation to reveal if differences in the patient journey and waiting time existed between different hospitals, specifically we applied the Lean technique of VSM to examine if hospitals with contrasting service organisation created differences in the timing of care for their patients. Our project included Public and Patient Involvement (PPI) to interpret the findings from the patients’ perspective.

## Methods

A VSM study of potential medical admissions was undertaken at four hospitals in South West England. The four hospitals were representative of UK acute hospitals, but were specifically selected to provide contrasting approaches to organising emergency medical care. Hospitals varied not only in their access routes and staffing configurations, but also in how they organised tests and results, patient seating areas and input from specialist care teams. These extra factors provided the context in which our patient data was collected. The main variations in care arrangements observed are described in Table 1. Further details of the hospitals characteristics are published elsewhere [16].

**Table 1:** Characteristics of care model which vary between sites, (✓ = present, x = absent)

Characteristics/Site	Hospital A	Hospital B	Hospital C	Hospital D
Catchment population size (approx.)	450,000	350,000	612,000	500,000
New ED attendances (annual approx.)	95,000	90,000	75,000	70,000
Conversion rate of A&E attendance to admission (range %)	26–34	22–28	30–36	30–40
Care model variations				
Innovation in the use of experienced clinical input	General practitioners	Acute physicians	Emergency medicine	Traditional approach
Use “Single point of entry”. All patients enter through ED	x	x	✓ by design	✓ by default
Automatic transfer of blood samples to lab	x	✓	✓	✓
Automatic test notification: blood results	x	x	x	✓
Barriers to prompt discharge (dispersed geographical population)	x	x	x	✓
Elderly assessment teams	x	✓	✓	✓
Discharge waiting area used	✓	x	✓	x
Medical/nursing routinely assist patient transfers (i.e. not relying on porters)	x	✓	✓	x
Clinical decision unit (CDU) or equivalent (an ‘off the clock’ area)	✓	x	x	✓

At each hospital there were notable features that influenced the patient care pathway, these were identified as:

Hospital A used an Acute GP service, where General Practitioners, located within the hospital setting, were able to advise community GPs by telephone, see some patients requiring same day treatment or hospital diagnostics, or admit non-ambulatory patients to a medical assessment unit.

Hospital B had a Medical Triage Unit (MTU) where GPs could refer patients directly if their condition was not life threatening.

Hospital C applied an innovative single entry portal. All non-scheduled patients, whether referred by a GP or not, attended the ED and were initially seen by their ED staff.

Hospital D was selected as a “control”, reflecting more traditional models of care where access is managed via the medical on-call team. Emergency patients were seen by the ED team, whereas GP referred patients arrived through ED but were seen by the medical team.

Despite these differences there were also similarities; all had set-up ambulatory care provision, and their proportions of unplanned admissions ranged from 22 to 40%. They all embraced the notion of early senior input – although implemented it in different ways. Hospital Episode Statistics (HES) data showed that during 2009–2013 emergency admission rates were rising for hospitals B-D (0.4–1.9% per annum), but had fallen for hospital A (−2.6% per annum). At all hospitals, service improvements continued to be implemented and developed during the course of our study.

### Participants

The study recruited participants from the ED or alternative routes (Acute GPs, MTU), representative of patients for whom the clinical decision makers were not immediately certain if admission or discharge was the best option. Participants were aged 18 or older, with cardio-respiratory symptoms or a presentation that was considered typical of older age (≥60 years of age with medical and/or social complexity). In preliminary work, these two groups were identified as often posing difficult decisions whether to admit or discharge. Patients were also required to be able and willing to consent to the study. Hospital staff identified a convenience sample of patients for possible inclusion, following which the researchers obtained informed consent.

### Data collection

Researchers recorded what was happening for each patient, minute by minute, from time of arrival until leaving the department, defined as patient journey time. The research team initially worked in pairs to collect data to ensure consistence in recording practice, remaining with the patients for their visit duration. Patients were also asked for basic information about reasons for attendance and expectations of care. All timing data were collected from the patient’s viewpoint, with particular attention paid to care activities and times where no observable activity was occurring. Clinical staff gave some additional information on reasons for waiting or details about the patient’s care.

Data collection was timed to coincide with a peak in patient attendance numbers [17] and departmental staffing levels. As such, this offered the maximum opportunity to recruit patients to the study and follow their care until the discharge or admission decision was reached. Patient attendance levels rose from 9 am, increasing through lunchtime and into the early afternoon. Patients were recruited at all sites between 9 am and 3 pm. Patients seen outside daytime hours and at weekends were not included in this study.

### Analysis

Traditionally VSM has been used as an iterative process to enhance organisational efficiency by reducing or eliminating waste; however, for our project rather than concentrate on the value stream map as an end in itself, we used the timing data to construct a cross-site comparison of patient care. The study focussed the VSM exercise on assessing patient pathway variation between sites, using the findings to interpret potential causality.

The VSM analysis examined patient journeys, during which the ratio of activity to waiting time was determined. Activity observed was predominantly patient contact time or an action related to their care that was seen or known to the patient (i.e. undergoing an x-ray); conforming to the Lean concept of a value activity. The Lean concept of waiting as a waste does not necessarily translate directly to the healthcare setting, where waiting may be necessary to accurately assess a patient’s condition. Therefore, waiting was recorded and classified as known or unknown reasons. For known wait reasons the most common were compared between sites.

To understand where waits were occurring, from the patient’s perspective, they were attributed within key timeframes in the patient pathway:

1. between arrival and assessment by a doctor
2. after assessment but before a final decision to admit or discharge was made
3. after the admit/discharge decision until leaving the department.

These process points were used as they applied to every patient and occurred in a specific order. Occasionally, a final decision was made when the patient was first assessed by a doctor, although generally, a working decision was made during assessment; diagnostic results confirmed the final decision.

### PPI and Stakeholder input

The study team conducted four workshops over the course of the project; an initial, mid-project, final and PPI specific workshop. Stakeholders from each site together with the PPI group attended all project workshops, during one of which they assisted the researchers to construct Value Stream Maps. These maps were predominantly built upon knowledge from senior ED consultants, they were refined following further feedback from a wider variety of staff on-site during face-to-face conversations with the research team.

Public and Patient Involvement (PPI) was included throughout the study, with the group contributing to discussions in all workshops [18]. Our PPI comprised of individuals brought together by the local CLAHRC (Collaboration for Leadership in Applied Health Research and Care) to comment and advise on health research projects within their region [19].

Once preliminary findings were available, the PPI specific workshop was organised to seek patient and public comments on data that had been collected and on initial research analysis. The workshop included PPI only to encourage maximum engagement from individuals. The PPI workshop data was fully transcribed and interrogated for themes that offered insight into the ‘meaning’ of our preliminary findings. We also noted any comments that spontaneously arose within the group to feedback into our analysis, reviewing raw data for confirmation of the views. In this way the PPI comments informed the interpretation of the study findings.

## Results

Between April and August 2014, 108 individuals were recruited. Twelve patients declined and four were excluded (their medical condition affected their capacity to consent), giving an uptake rate of 87%. In total 25,432 min of patient time were observed, containing 4098 separate episodes of patient care or waiting time.

Participant characteristics are detailed in Table 2. These included a wide age range, with an equal representation of both genders but little variation in ethnicity. The complexity of patient pathways is indicated by the range of care process steps and numbers of different staff contacts per patient.

**Table 2:** Characteristics of study participants

Characteristics of participants	Hospital A	Hospital B	Hospital C	Hospital D	Total
Number of participants	30	30	24	24	108
Presentation					
Cardio-respiratory	19	15	16	14	64
Older age	11	15	8	10	44
Gender					
Female	15	16	12	12	55
Male	15	14	12	12	53
Age (range, median)	26–94, 65	18–93, 78	23–94, 70	47–99, 82	18–99, 76
Ethnicity					
White	29	30	24	24	107
BME	1	0	0	0	1
Characteristics of care process during patient journey					
A health professional referred the patient through the acute care route:					
No – ED only route	19	23	13	17	72
Yes – Medically expected route (which may include ED for some sites)	11	7	11	7	36
No. care/waiting episodes recorded per patient (range, median)	10–51, 26.5	12–72, 31.5	21–75, 41	26–88, 50	10–88, 34
No. different staff encountered per patient (range, median)	2–10, 4	2–21, 5	3–12, 7.5	6–13, 8.5	2–21, 6

### Overall journey time

**Fig. 1:**
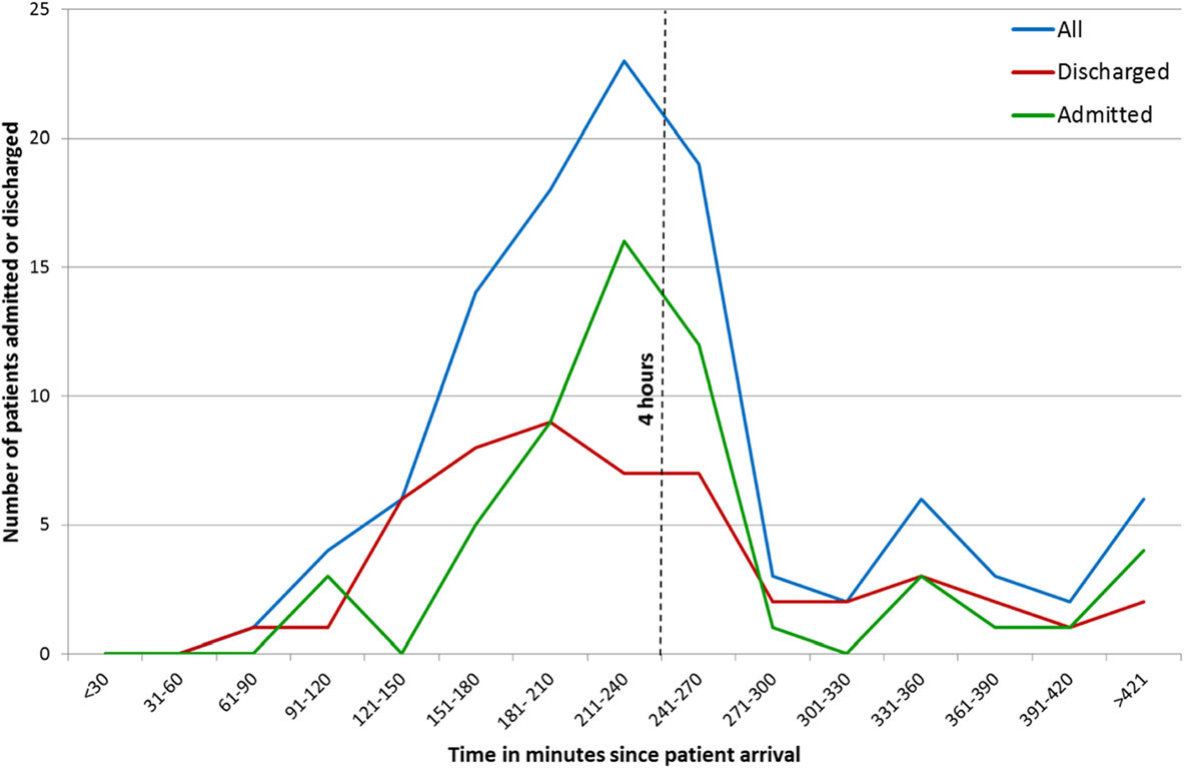
Acute patients admission and discharges by time of arrival

The median patient journey time was 223 min. The shortest time was 62 min (hospital B, MTU), the longest, 692 min (hospital A, ED using the CDU). Figure 1 illustrates the relationship between the 4-h standard and admissions/discharges occurring. Few decisions were made within the first hour, whereas admission and discharges rose between two and four hours, and thereafter declined rapidly.

**Fig. 2:**
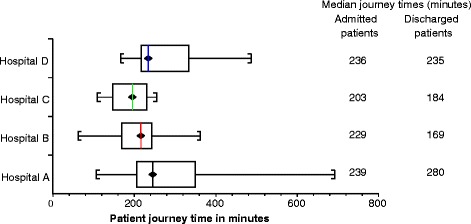
Median patient journey time with semi-interquartile range, by site

A summary of patient journey time by site (Fig. 2) showed little variation between median journey times, but did suggest more substantial differences in the range of journey times experienced by patients. The longer journey times at Hospital A were using the CDUs for patients awaiting test results, where ED staff remained responsible for the patient.

Dividing participants by discharge status, we found no consistent difference between sites. At hospital D there was no discernible difference in journey times for admitted or discharged patients. However, shorter journey times were observed for discharged patients at hospitals B and C, whereas the reverse occurred at hospital A.

### Differences between sites

**Fig. 3:**
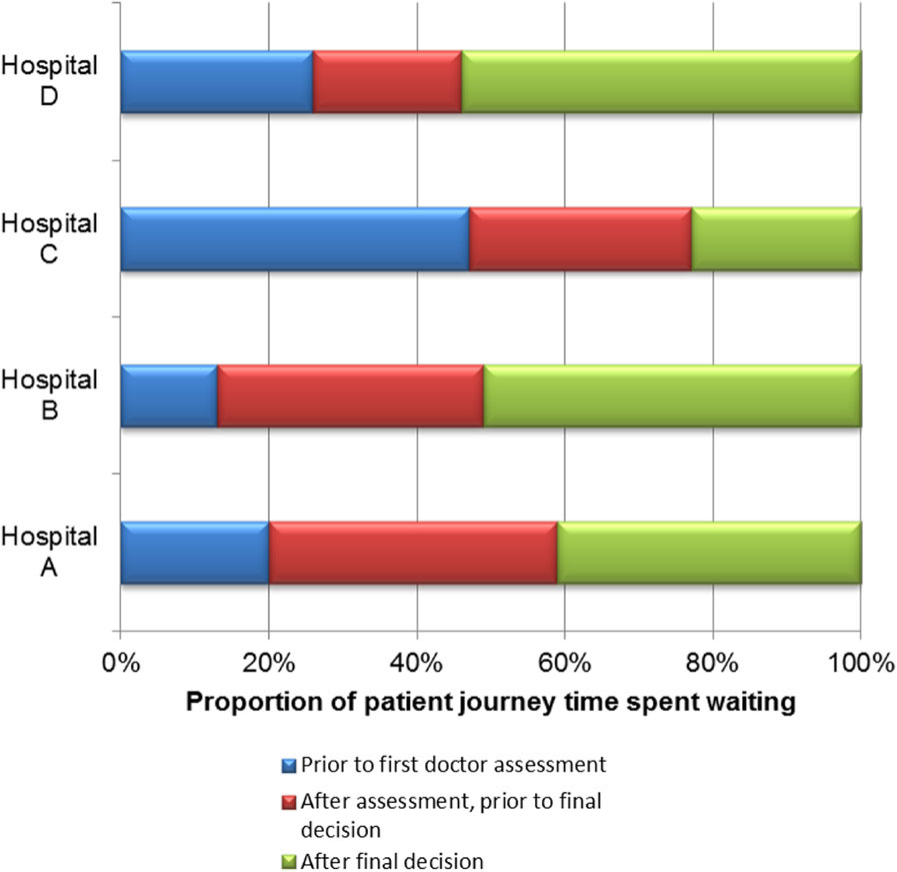
Stage of patient care where waiting occurred

To compare and contrast the differences between sites we summarised the stage at which waiting occurred (Fig. 3). We observed a clear variation in the stage where waiting time dominated the patient journey. Hospital C showed the greatest initial wait time, after arrival, but before being assessed. This site employed a single entry point, where all patients arrived through, and were assessed by, ED staff. Although the largest proportion of the waiting time occurred around arrival, absolute assessment and treatment times were amongst the shortest (Fig. 2). In contrast, hospital B had the shortest initial wait, but was similar to hospital D in having longer waits for patients to leave the department.

We found that patients often did not know why they were waiting. From the patient perspective, the reason for waiting was unknown for: 42, 42, 76 and 67% of total waiting time for hospitals A-D respectively. Given the nature of acute health care a degree of patient waiting for unknown reasons would be expected, and appears similar to the pattern of waiting stage observed – highest where a longer initial wait is present, before assessment or tests are underway.

### Reasons for waits

**Fig. 4:**
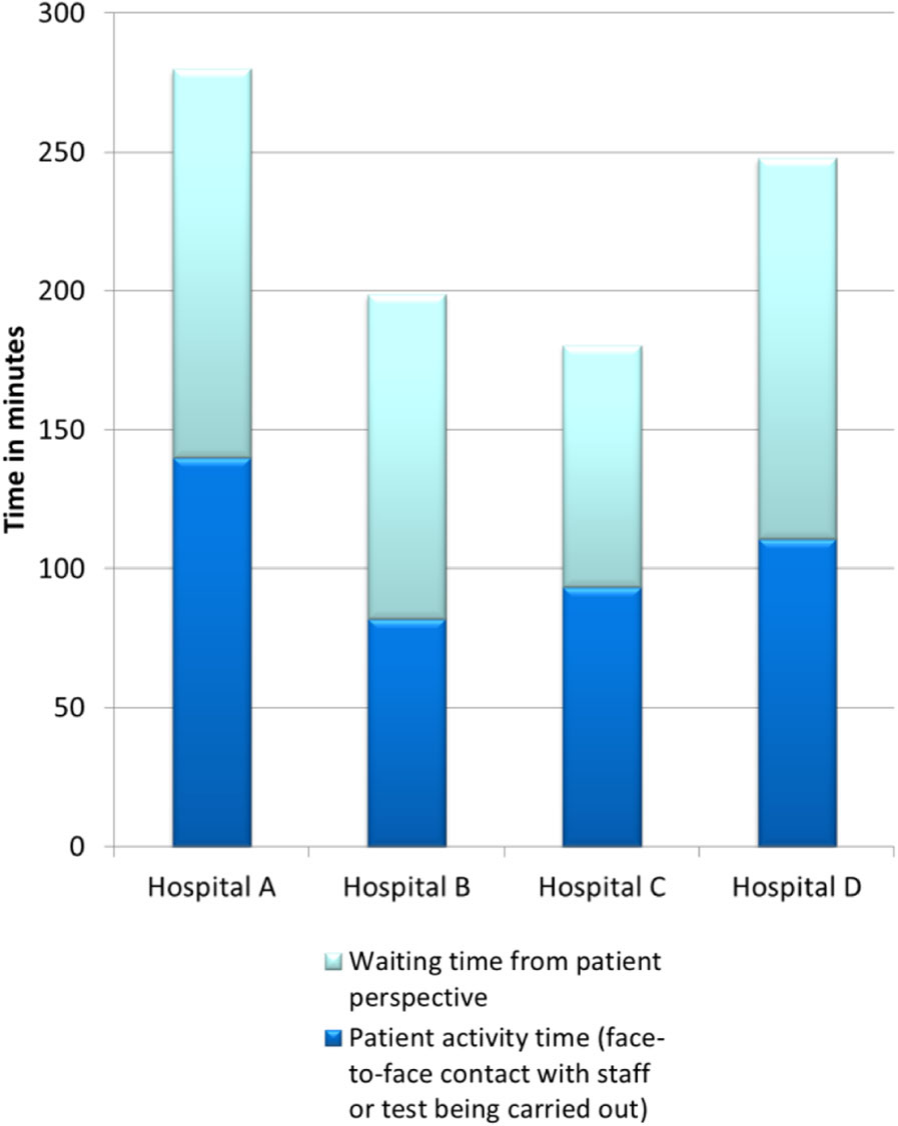
Patients’ experience of activity and waiting

Using the Ohno categorisation, our data was split between known and unknown waits and patient activity (Fig. 4). The overall recorded activity time ranged from 39 to 52% between sites, with an average of 45%. There was noticeably more activity time in total in Hospital A.

**Fig. 5:**
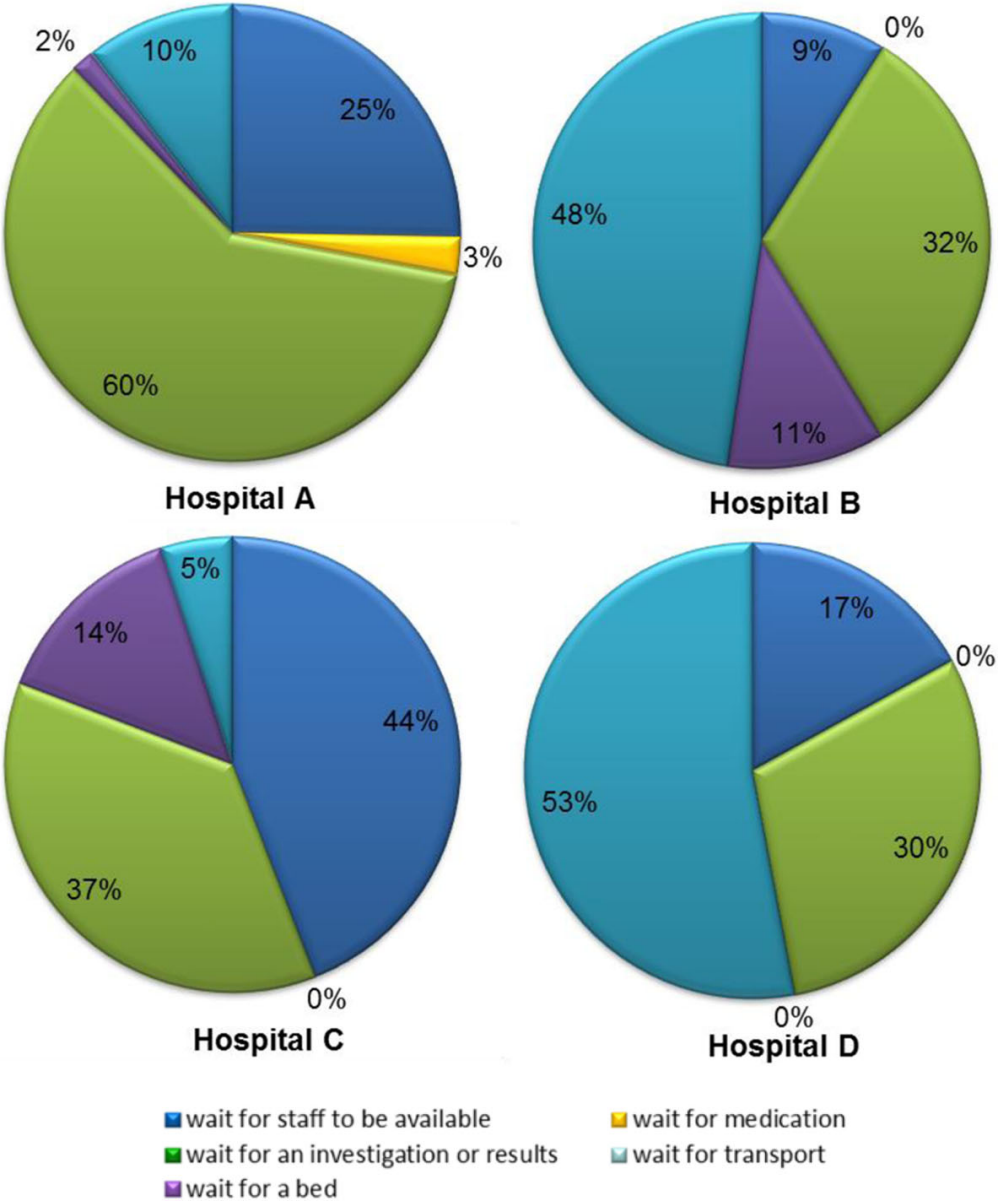
Types of known waiting from the patient’s perspective

Looking in more detail at the reason for observed waiting, Fig. 5 shows types of known wait from the patient’s perspective.

Hospital A differed from the other sites, having the longest proportion of waiting time (60%) attributed to investigations and test results. This site used a CDU for patients awaiting results. Where appropriate, they also streamed through an Acute GP service. This was the only site exhibiting a reduction in the rate of emergency admissions in recent years.

### Public and patient involvement workshop

The PPI workshop was attended by four patient representatives. Their comments were reflections on their own experience and that of accompanying relatives or friends through acute or emergency care. The group emphasised how waiting at different times during the visit might be experienced differently. Table 3 shows an excerpt from the workshop explaining some of their perspectives about waiting.

**Table 3:**
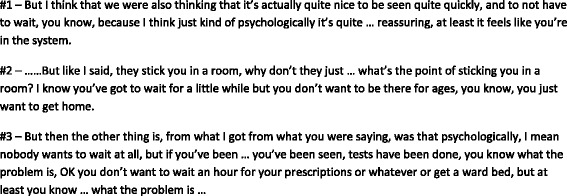
Excerpt from public and patient involvement workshop

Two main concepts affecting patient experience were identified from the workshop; time of day – long waits late in the evening were regarded unfavourably, as were long delays at the outset of the patient journey, before they were ‘in the system’.

## Discussion

Despite the need to recruit patients in a stressful, challenging environment, participation rates for this study were high, at almost 90%. The data from four sites allowed detailed comparison to be made in the time-course of patient care.

### Summary of main findings

We found that although a potential admission decision might be made early in the patient journey, a final decision and action point often did not occur until prompted by the 4-h standard, matching what has previously been captured in national data [20]. All sites showed the same admit/discharge actions clustering around 4 h, yet how each site organised their care model to respond to this driver varied.

At the hospital designed for single entry point entry, patients experienced longer initial delays. These may be in response to a greater number of undifferentiated patients seeking access through the same route. The alternative for patients was to go directly to a unit expecting them via a GP referral, freeing up resources in the ED for unscheduled patients. The PPI group commented that ‘not being in the system’ could cause significant anxieties. Delays to definitive assessment can also be associated with a greater level of clinical risk, if a serious condition is not recognised at an early stage. However, despite these anxieties, our data suggested that initial delays did not lead to a longer total journey time – rather the converse; on average patients spent less time in acute care at this site. We therefore conclude that while reduced patient waiting before assessment may alleviate anxiety, it does not necessarily result in a shorter overall patient journey for those with an unclear admission choice at presentation.

While patients were using acute care and emergency services they often had relatively little insight into the underlying reasons for waiting. For example, the PPI group reflected that waiting on portering services was unacceptable, yet did not question whether a test result could have been provided quicker if services were organised differently. There was an acceptance that blood test results would take time, however our data showed that this varied between sites, and systems such as automatic transfer of samples to the lab, or electronic notification when results become available, might influence how long patients spent awaiting these results.

At two sites over 50% of the average patient journey time occurred once the decision to admit or discharge was made, coinciding with waiting for beds (approximately 50% of known patient waits), or to leave the hospital. Neither of these sites used a patient discharge lounge. The combination of these two factors implied the existence of barriers to admitting and discharging their patients. From our data we cannot interpret whether delays associated with bed availability at these sites were caused by resource or system limitations, although we can suggest that addressing this final stage of the patient journey could result in improved patient journeys at these hospitals.

### Strengths and limitations

Our study modified the VSM approach to gather patient flow data from four contrasting hospital sites, highlighting where variation existed, to provide data that could assist service design and commissioners. An important limitation was in the number of variables that could be accommodated in the VSM model. The study attempted to limit this problem by selecting just two patient groups and only collecting data during usual daytime work hours. Therefore, the services were represented at peak efficiency and for two of the most important patient groups. The range of service models and factors influencing patient pathways may limit the generalizability of these findings to other hospitals, which would need to evaluate their own patient waits in order to most effectively direct their resources for care improvement. We did not look specifically at the phenomenon of crowding and “exit block”, which are known to have important impacts on access to acute hospital beds [21], as this represents only one part of the overall patient journey that we examined, and has already been well documented by other authors. The study benefitted from ongoing integration of PPI and inclusion of user feedback to interpret the findings.

### Comparison with literature

During the past decade Lean techniques have been applied in EDs and other healthcare settings. Positive results have been published on instigating and maintaining improvements in healthcare, efficiency and patient satisfaction [22]. However, only one study has considered more than site; this study observed the effects of different uses of Lean across four sites over time [23]. Ours is the first study applying a coherent VSM approach to multiple sites simultaneously to compare patient pathways.

Our findings, on differences in patient journey and interpretation of waiting, resonate with others in the field. Holden and Smart [3] found that patients ranked waiting as the most important factor during their visit. In a systematic review Taylor and Benger [4] drew attention to the importance of patients perceived waiting times and emphasised the relevance of addressing the issues of patient waiting and timely communication to manage expectations. Nairn’s [24] review of patient experience linked waiting and satisfaction, but also cautioned that nuances of quality and care are not captured through quantitative focus; a finding we addressed by inclusion of PPI.

The differences in waiting patterns we observed between sites highlights a variation which is potentially modifiable. Although it is hard to achieve balance between demand and capacity at certain times [25, 26], Ortiga [15] showed that variation in ED attendance could be successfully managed, thus directly addressing prolonged waiting. This excessive waiting, a feature identified as creating a culture where staff reacted with frustration, shame and eventually resignation, could be modified by changing the patient experience [14]; Burstrom suggested that once patient flow has been optimised, staff address patient frustration by calming and informing them about waiting times [13]. However, our data implies that patient flow is not yet optimised, because although total journey time meets the 4-h target, there is still considerable variability between sites in elements of the journey (e.g. early assessment, automatic test notification and post discharge-decision waiting time).

We found that sites without a discharge/decision unit have a greater proportion of patient wait times after the decision to admit or discharge has been made. Therefore, provision of discharge, or seated, areas as suggested in the Department of Health Planning and design guidance for EDs [27], may offer one alternative for improving the patient journey through acute care.

At some sites 50% of known waiting was for bed availability, corroborating Higginson’s observations [5] that patient flow and crowding problems are often caused by issues outside the control of ED. Similarly, while Ng [7] found that applying Lean thinking could create improvements in ED performance, if this were not applied to the whole organisation, waits and blockages may simply be moved elsewhere, and bed blocking due to high hospital bed occupancy still dominates. Although VSM analysis can pinpoint inefficiencies within the patient journey, achieving more substantial improvement requires consideration of the whole healthcare context [28]. Further research may explore system modifications that could reduce waits and improve patient experience by looking at the wider system context and across functional divides, such as departmental boundaries.

## Conclusions

VSM analysis of patient journeys across four different EDs, coupled with PPI interpretation, highlighted how the 4-h standard dominates the timing of admit/discharge decisions, but also gave insight into how different service models can affect the patient’s experience. We have shown how different service models affect the way in which patients’ waiting times are distributed throughout their hospital attendance and that there were substantial variations between these four different hospitals. Based on these data, arrangements that could reduce patient waiting include automated specimen transfer and result notification systems to minimise diagnostic delay, and ambulatory discharge/clinical decision areas to accommodate patients undergoing periods of essential clinical observation, awaiting tests or test results, or for transport home. In practical terms, hospitals wanting to reduce the patient journey time through their EDs should examine their own patient waiting patterns to determine where resources can be most effectively used. In circumstances when waiting is necessary, either for clinical diagnostic reasons, or at times of high demand, better explanation of reasons for waiting and likely wait times, especially before a patient sees a doctor, is likely to improve patient experience.

## Abbreviations

BME: Black/minority ethnic
CDU: Clinical decision unit
CLAHRC: Collaboration for leadership in applied health research and care
ED: Emergency department
HES: Hospital episode statistics
MTU: Medical triage unit
PPI: Public and patient involvement
REC: Research ethics committee
VSM: Value stream mapping
